# Supplementary material for: Exploring the causes of COPD misdiagnosis in primary care: A mixed methods study
Source: PLoS One. 2024 Mar 6;19(3):e0298432. doi: 10.1371/journal.pone.0298432 (PMC10917297; doi:10.1371/journal.pone.0298432)
Supplement: S1 File — (DOCX) [file pone.0298432.s001.docx]

## Supplement file 1 – Topic guide

**Topic 1: Diagnosing COPD (only applies to HCPs)**

**-Aim is to obtain information regarding clinician views on the diagnostic process for COPD**

*Prompt questions:*

*“How do you normally go about diagnosing a patient with COPD?”*

*“What normally prompts you to investigate a patient for COPD?”*

*“How often do you see patients with COPD?”*

*“How often do you diagnose patients with COPD?”*

*“Do you feel confident making a diagnosis of COPD?”*

*“Who do you think should be making a diagnosis of COPD and why? (GP or Specialists)”*

**Topic 2: Spirometry (only applies to HCPs)**

**-Aim is to obtain clinician views on spirometry use**

*Prompt questions:*

*“Do you feel confident using a spirometer?”*

*“Do you feel comfortable interpreting a spirometry reading?”*

*“How do you feel about the use of spirometry in COPD?”*

*“If spirometry was no longer a QOF outcome would you still perform spirometry?” -****GPs ONLY***

*“Do you think there is a difference between spirometry completed in GP vs Hospital? If so what do you think the difference is?”*

*“Tell me what you know of using lower limit of normal to assist in diagnoses when assessing elderly patients?”*

**Topic 3: Exploring differentials (only applies to HCPs)**

**-Aim is to obtain clinician perspective regarding how they consider alternative diagnoses**

*Prompt questions:*

*“When diagnosing patients with COPD what alternative diagnoses do you consider?”*

*“How do you investigate for alternative diagnoses?”*

*“Do you think it is important to consider alternative diagnoses to COPD?”*

*“What factors influence whether you investigate for alternative diagnoses?”*

**Topic 4: COPD misdiagnosis (only applies to HCPs)**

**-Aim is to obtain the clinicians opinion on misdiagnosis**

*Prompt questions:*

*“Are you aware of any issues regarding misdiagnosis of COPD?”*

“What do you think are the causes of patients being misdiagnosed/mislabelled with COPD?”

“Do you think COPD misdiagnosis is a serious issue? And why?”
“Have you taken any actions in the past to reduce the number of patients misdiagnosed with COPD? if so what actions?”

**Topic 5: Solution to misdiagnosis (only applies to HCPs)
-Aim is to identify how clinicians feel misdiagnosis can be prevented**

Prompt questions:
“Do you have any thoughts on how misdiagnosis with COPD can be prevented?”
“Do you feel national standards regarding spirometry will help prevent misdiagnosis?”
“Do you think further training for healthcare professionals would help?”
“Is COPD too complex to be diagnosed in primary care? And why?”

**Topic 6: Misdiagnosis (only applies to misdiagnosed patients)**

**-Aim is to explore patients’ feelings and thoughts about being misdiagnosed**

*Prompt questions:*

*“How do you feel now that your diagnosis is no longer COPD?”*

*“Do you have any concerns about being diagnosed incorrectly?”*

*“How has this (misdiagnosis) impacted you if at all?”*
