# Supplementary material for: Exploring the causes of COPD misdiagnosis in primary care: A mixed methods study
Source: PLoS One. 2024 Mar 6;19(3):e0298432. doi: 10.1371/journal.pone.0298432 (PMC10917297; doi:10.1371/journal.pone.0298432)
Supplement: S2 File — (DOCX) [file pone.0298432.s002.docx]

**Supplement File 2 – Coding framework**

| Code | Definition |
| --- | --- |
| Preventing COPD misdiagnosis | Discusses interventions and thoughts to avoid COPD misdiagnosis |
| Managing COPD misdiagnosis | Discusses thoughts on how to treat misdiagnosed patients |
| Causes of COPD misdiagnosis | Discusses perceived factors leading to misdiagnosis |
| Impact of COPD misdiagnosis | Perceived short and long-term effects of COPD misdiagnosis |
| Patient relief | Expresses relief for patients |
| Spirometry experience | Discusses experience with spirometry |
| Spirometry interpretation | Discusses any aspect of interpreting spirometry results |
| Spirometry- Patient technique | Discusses technique of performing spirometry from patient perspective |
| Spirometry procedure | Discusses the process of performing spirometry |
| Spirometry quality | Expresses thoughts on quality of any aspect of spirometry testing |
| Spirometry resources | Discusses availability of equipment for spirometry |
| Spirometry skills | Expresses thoughts around HCP skills with any aspect of spirometry |
| Spirometry training | Discusses thoughts about training for any aspect of spirometry testing |
| Impact of COVID – Spirometry | Thoughts about perceived impact of pandemic on spirometry provision |
| Use of spirometry | Discusses thoughts on utilising spirometry |
| Access to spirometry | Thoughts about ability to obtain spirometry testing or results |
| Diagnostic differentials | Discusses thoughts on alternative diagnoses when diagnosing COPD |
| Diagnosing COPD | Thoughts about any aspect of diagnosing COPD |
