# Supplementary material for: Exploring the causes of COPD misdiagnosis in primary care: A mixed methods study
Source: PLoS One. 2024 Mar 6;19(3):e0298432. doi: 10.1371/journal.pone.0298432 (PMC10917297; doi:10.1371/journal.pone.0298432)
Supplement: S3 File — (DOCX) [file pone.0298432.s003.docx]

**Supplement File 3 - Category summaries**

**Category 1: Misdiagnosing COPD**

Definition

The perceived processes involved in misdiagnosing patients with COPD and the outcome of such misdiagnoses.

**Codes:**

“Preventing COPD misdiagnosis”, “Managing COPD misdiagnosis”, “Causes of COPD misdiagnosis”, “Impact of COPD misdiagnosis”, “Patient relief”

Summary of data

**Misdiagnosis pathway**:

Sense of anchor bias, whereby a historical diagnosis of COPD without spirometric evidence would lead to tunnel vision and prevent consideration of alternative diagnoses in patients already diagnosed with COPD*.* ***“And the issue, I think most of the patients that have that from here were historic patients that were been diagnosed ages ago without correct testing, or they were admitted to hospital and they get a TTO saying exacerbation of COPD, and I go they haven't got COPD. But of course, when that happened 5-10 years ago, it was just coded.... And that code just appears.” – SIN008 GP.*** Errors in historical diagnoses attributed to diagnoses being based on clinical findings and symptoms. ***"Okay, some of them may have been historically misdiagnosed. We only started doing spirometry about five years ago, I think or something like this. I'm certain about it. Because I was diagnosing COPD on history in the past.. smoker who's got recurrent infections, wheezing etc and lots of things over there. And I'm talking about more than 10 years ago, or even 15 years ago, spirometry started coming in."- SIN007 GP.*** Secondary care participants corroborated the perceptions of primary care ***“they do presumed COPD, but I'm not confident that it always the surgeries go back to reassess that. I think they just once they mentioned COPD on their read codes.... it's hard, isn't it to pick up where the diagnosis and when the diagnosis occurred” -SIN013 Respiratory Nurse.*** Secondary care participants focused on the lack of spirometry use in primary care leading to patients being misdiagnosed initially ***“people referred up to the clinic or they come into hospital with a label of COPD. There's no spirometry on the system, or they've come in with breathlessness and someone's given them a diagnosis of COPD. And actually when they come back to outpatients they've got normal spirometry.”- SIN023 Respiratory Consultant.***

Participants focused on regular review and challenging historical COPD diagnoses as a method of reducing the extent of misdiagnosis in primary care. ***“not just making a diagnosis, but making sure they have regular reviews, even those that don't seem to be having a frequent exacerbations. Perhaps having a standard whereby you review them once a year, at the very minimum and when you review them, it could very well be that it comes to light then that the diagnosis wasn't made correctly”- SIN022.*** Specialist support and education was perceived as an important intervention to assist in correcting misdiagnosed cases and preventing future misdiagnosis. ***“I think it's working together primary care, secondary clinicians, you know, keeping up to date having someone here helps in that in that we're aware more. Therefore, we're actually looking at where, perhaps more educated on looking at for the right symptoms and signs of COPD and asthma and distinguishing between the two.”- SIN005 GP.***

The perceived misdiagnosis pathway therefore being: Initial misdiagnosis due to lack of spirometry 🡪 no diagnostic review in primary care 🡪 persistent population of misdiagnosed patients in primary care.

**Misdiagnosis outcome:**

Patient participants focused on the sense of relief for not having COPD, the perception of COPD was often negative and assumed to limit their life ***“Well, COPD that's where your tubes are shrinking and you know and I thought that was it .. the beginning of the end if you get what I mean. Now we know that it's not, it's taken that bit of weight off my mind”- IN16038 Patient.*** HCP participants focused their concerns for the impact misdiagnosis had on the patient’s health, mental and social wellbeing ***“it can impact on people's life insurance, travel insurance. Have they been taking steroids that perhaps they didn't need to take? Has that put them at higher risk of getting pneumonia? You know, you know, have been given lots of courses of steroids that, that didn't need putting them at risk of osteoporosis, you know, so suppose and I don't know, maybe psychologically, the thing, they've got this sort of, not death sentence, but it's a long term condition, isn't it that's progressively gets worse over time. You don't know whether that perhaps has a psychological effect on some people and, you know, and, and people's occupation, if they if they put it down to they can't do the job because they've got COPD, whereas actually, it could be something else.”- SIN021 ACP.***

**Deviant case:**

One GP (SIN016) focused on misdiagnosis being inevitable and that it was “part and parcel” of being a GP, but the focus should be to ensure quality of life is improved not getting the label correct.

**Final points:**

Misdiagnosis is prevalent in primary care due to inadequacies in the diagnostic review process and historical diagnoses often lead to anchor bias. Historical diagnoses were often made on clinical grounds only without spirometry. Patients often felt relief when told they do not have COPD, and HCPs were concerned that misdiagnosis has the potential to have a significant impact on a patients life. Combination of specialist input and diagnosis review were perceived as an intervention to reduce misdiagnosis in primary care.

**Category 2: Spirometry**

Definition

Thoughts or perceptions surrounding spirometry in primary care.

**Codes:**

“Spirometry experience”, “Spirometry interpretation”, “Spirometry- Patient technique”, “Spirometry procedure”, “Spirometry quality”, “Spirometry resources”, “Spirometry skills”, “Spirometry training”, “Impact of COVID – Spirometry”, “Use of spirometry”, “Access to spirometry”.

Summary of data

**Role of spirometry in primary care:**

Spirometry was perceived as a useful tool to help with the management of patients with COPD in primary care ***“You know, to have a spirometry done every year or two. And certainly, if there's a deterioration in symptoms, you know, doing another one at that point, just so that we can see clearly what is happening (…) So yeah, I quite like to be able to look back over several different readings and see how it's changed over a period of time”* *-SIN020 GP.*** However, there were opposing views regarding the necessity of spirometry to diagnose COPD. It was perceived by some as essential ***“I say that COPD is a physiological diagnosis, you wouldn't treat blood pressure without first... you wouldn't treat hypertension without first measuring the blood pressure. So you need to do the diagnostic test which is spirometry” -SIN023 Respiratory Consultant.*** However, it was also perceived as a “tick-box exercise”, ***“if it's easy, you almost don't need this spirometry like you look at them and clinically, you make the diagnosis and you are almost certain, and you're like, "Yeah, you've got COPD" and you do spirometry to tick a box”*** ***-SIN009*** ***GP***. Significant clinical history alone was perceived as sufficient to diagnose COPD, but there was an appreciation that spirometry was useful when there was diagnostic doubt ***“with COPD, you've got people that might have a mixed mixture, and there are other things that could be going on, so I think you really need to have the spirometry to determine what's going on.” -SIN022 GP.***

Within primary care, performing and interpreting spirometry was perceived as part of the nursing role, with nurses discussing their experience doing spirometry and doctors describing referring patients to nurses to have spirometry ***“..refer this patient to the nurse to get a spirometry done. And then based on the spirometry, we would see what the FEV1, and then the ratio and then we decide if it is COPD”*** ***-SIN015***. Whereas the decision to investigate with spirometry was perceived as a doctor’s responsibility ***“But we only do that if that comes from the GP. So, it's the GP that decides whether we do spiros, on which patients and when” -SIN010 Practice nurse*** as was labelling the patient with COPD “***the nurse because of their expertise might actually be able to help the GP or the GP trainee, because we're a training practice, might help them to interpret the results (…) but in terms of putting their firm diagnosis on the record, it will be done to a doctor” -SIN022 GP***. Annual COPD reviews were completed by practice nurses and as a result it was perceived they had greater experience with spirometry and COPD ***“I am confident but not as good as my nurse. My practice nurse in surgery, she's, she's excellent at reading this spirometry; a lot better than me” -SIN015 GP.***

However, there were doubts amongst secondary care HCPs regarding the quality of spirometry results obtained in primary care ***“in terms of the spirometry, if it's been done in primary care, then I don't think they get I'm not confident that they have reproducible traces I'm not confident that it's done when they are, you know, at least four to six weeks post exacerbation. I'm not confident that they will out any alternative diagnoses with reversibility, like asthma, just because of the timing that it takes for the reversibility testing” -SIN013 Respiratory nurse***.

**Enabling factors and barriers**:

Participants focused on the training and accreditation as an enabling factor, knowing that nurses had been trained or had attended courses led to GPs having greater confidence in their ability and felt comfortable seeking their advice with COPD. ***“she was actually doing another course to update herself. And she's done the exam as well. And she passed it. So she's the one who's up to speed on spirometry, probably, actually, to be honest, I would say much more than I am. I have to sort of go back and look up guidelines and remember how to, to interpret the results, actually, because she does so much of it in practice”*** ***-SIN018 GP***.

Having “in-house” spirometry was deemed as cost-effective and easy for patients, thus was perceived as an enabling factor to its use in primary care ***“if we just provide it in house well then we're not paying for it.. we get the money for providing the service. But it also means that patients get it quicker because we can just book it rather than referring and waiting however long it takes to get it” -SIN008 GP***. Easy access to community hubs were also seen as an enabling factor as they were deemed cost effective ***“number of COPD patients that probably no more than 15 I think. Thus, to provide the equipment, run the service is financially not viable (…) So we entered into a contract with a community respiratory team who, in return for a charge, they would do it because they have clinic not far from us” -SIN017 GP***. One participant did suggest a re-organisation of services such that spirometry was provided in hubs to maintain skills and improve access for all patients ***“So with the GPs choosing their services, you should have, say one of the bigger practices where you've got a GP who's got big respiratory interest. And, you know, he's trained and confident with the diagnosis. And they've got someone who accurately performs the spirometry correctly, even undertaken the ARTP course. And that those surgeries can link in with them. And they have an arrangement to send their patients for spirometry, to either rule it in or out” -SIN013 Respiratory nurse***. The COVID 19 pandemic was perceived as the main barrier to spirometry through preventing training courses and leading to deskilling amongst primary care HCPs ***“I was supposed to start the training and haven't started doing anything, because we've had to stopped doing as much face to face. And we still haven't caught up with the COPDs yet” -SIN001 Practice nurse***. This was perceived to have a potential knock-on effect on the integrity of the diagnoses made based on clinical history alone ***“Obviously, it's effected diagnosis for new patients or patients with new symptoms. Because there's no spirometry” -SIN013 Respiratory nurse.***

**Final points:**

The role of spirometry was perceived as one to assist in the management of patients with COPD, which is primarily the role of the practice nurses. Spirometry was perceived as essential to make a COPD diagnosis by some HCPs but also seen as a confirmation investigation once diagnosis made with clinical history, but appreciated its role with difficult to diagnose cases. Having adequate training and easy access to spirometry were key enabling factors to its use, however, COVID 19 was perceived as the predominant barrier to its use, with concerns about potential long term impacts.

**Category 3: Diagnosing COPD**

Definition

Thoughts and perceptions surrounding how and where COPD should be diagnosed and difficulties differentiating COPD from other pathologies.

**Codes:**

“Diagnostic differentials” and “Diagnosing COPD”

Summary of data

**Generalist Vs Specialists:**

Diagnosing patients with COPD was perceived as an activity best suited to a primary care setting with primary care clinicians taking the lead due to perceived limited access in secondary care as well as a perception of regular interactions with patients ***“both can diagnose. So if specialists are, if only a diagnosis can be made from secondary care, you might increase your ability to diagnose, but you will have really long waiting lists and wait for that will be much, much longer” -SIN016 GP.*** Participants felt that the responsibility of diagnosing COPD fell with the primary care clinicians rather than secondary care specialists ***“Umm general practitioners, nursing staff, I think practice nurses. And I don't necessarily think this is just something that specialists should do, it should be something we pick up in primary care. And because we're the people who tend to see patients more regularly, and so if they're coming to us with recurrent symptoms of spastic chest infections, and coughing, wheezing, then we should look forward to making that diagnosis” -SIN018 GP*** Participants perceived primary care HCPs as being capable of diagnosing COPD, with appropriate guidance ***“So mentally, I think I think it's nice to have a specialist who can focus on it. And then we can have that discussion its useful to have a with our situation healthcare assistant who is very interested in COPD and chest medicine, and then GP with ongoing kind of interest and then having the specialist so we kind of bounce the ideas that description... that is what a multidisciplinary team is. And I think I think the days of having have one I'm the GP and I made a diagnoses, it doesn't hold you know.. we should discuss it in a multidisciplinary team kind of way” -SIN012 GP.***

**Asthma Vs COPD:**

Participants focused on the difficult of differentiating COPD from asthma due to similar symptoms ***“I feel that sometimes, they come in being treated for asthma, and it's not relieved from the inhalers that they're on is not relieving. And you know that they're a smoker, and you just feel is it asthma or is it COPD, you know have they got there. Whereas then when we've queried it with the doctors, I think they haven't quite been certain themselves at the time”*** ***-SIN010 Practice nurse***. Participants perceived specialists as having more experience with respiratory conditions, thus enabling them to differentiate easily ***“I think that occasionally COPD is difficult to diagnose (…) I'm suspecting it COPD or possibly asthma or possibly overlap, and I don't either have the knowledge for it or, or is my suspicion, I definitely think that my senior My, my, my colleagues who are specialists in it should give me feedback, they should see that person they should advise me” -SIN007 GP.*** COPD was perceived as the default diagnosis when unsure of causes of breathlessness in smokers and ex-smokers ***“it does kind of make us go into a one track mind. So if someone presents with breathing difficulty or whatever, we would probably just give them antibiotics and steroids and just let them get on their way and review them in a week and a half or two weeks. But there are patients who aren't that clear cut and actually, I think we do them a bit of a disservice by just thinking they have COPD, they can get other things. And that treatment may be slightly different” -SIN018 GP.*** Difficulty differentiating COPD from other respiratory conditions was perceived as a factor leading to misdiagnosis ***“Ah yeah, we've had that lots of times, where we think the patient is, for example, asthmatic, and we've been treating him for asthma. And then we had MDTs, or consultants, have gone into hospital and the diagnosis had been COPD all along. So yes, we've had a few of those over the years where we've obviously labelled the person has one condition, but they've got something else” -SIN015 GP.***

**Final points:**

Participants felt strongly about primary care being the idle setting and GPs being best suited to diagnosing patients with COPD, however, appreciated specialist input is needed with difficult cases. Difficulty predominantly focused on differentiating asthma from COPD, where specialist experience and knowledge was perceived to ease that confusion.
