# Supplementary material for: Exploring the causes of COPD misdiagnosis in primary care: A mixed methods study
Source: PLoS One. 2024 Mar 6;19(3):e0298432. doi: 10.1371/journal.pone.0298432 (PMC10917297; doi:10.1371/journal.pone.0298432)
Supplement: S4 File — (DOCX) [file pone.0298432.s004.docx]

**Supplement file 4 – Thematic map**

Asthma Vs COPD?

Negative impact of COPD label on patient care

Negative connotations linked to having COPD label

Diagnosing COPD is a GP’s role

Tunnel vision from historical diagnosis

Specialists have more experience

Mixed views on usefulness of spirometry

Ongoing exposure needed to maintain spirometry skills

Reduced spirometry due to COVID19

Lack of spirometry 🡪 No diagnostic review 🡪 Persistent population of misdiagnosed patients.

Reluctance to challenge historical misdiagnosis

Historically no spirometry available

Nurses do spirometry, doctors read spirometry

Historically clinical evidence enough for diagnosis

Challenges in differential diagnosis
