# Supplementary material for: Exploring the causes of COPD misdiagnosis in primary care: A mixed methods study
Source: PLoS One. 2024 Mar 6;19(3):e0298432. doi: 10.1371/journal.pone.0298432 (PMC10917297; doi:10.1371/journal.pone.0298432)
Supplement: Attachment — Submitted filename: Response to reviewers.docx [file pone.0298432.s005.docx]

Dear Editors,

We would like to thank the Editors and Reviewer for their valuable feedback on our manuscript titled “Exploring the causes of COPD misdiagnosis in primary care: A mixed methods study”.

Please find below our responses to comments from the Editor:

1. As recommended, we have edited our manuscript to meet the journal’s style requirements.
2. We have amended the manuscript to include that all participants gave informed written consent.
3. Our updated competing interests includes the statement “This does not alter our adherence to PLOS ONE policies on sharing data and materials.”.
4. We have included the minimal data set as a supplement in supplement files 2, 3 and 4.
5. We have amended the manuscript to include the ethics statement in the Methods section.

Please find below our responses to the Reviewers comments:

1. We have completed and attached the COREQ checklist as recommended.
2. Purposeful sampling was conducted through assessing the demographics of potential participants and identifying participants to approach to ensure heterogeneity. No software or tools were used to support this process.
3. Theoretical saturation was determined based on the review of ideas emerging from consecutive interviews, once the lead researcher felt no new ideas were emerging the possibility of saturation was discussed within the research team and once all researchers in the team were in agreement that saturation had been reached further recruitment ceased. The research team consisted of 3 clinical researchers, 3 non-clinical researchers and a medical student.
4. Patient representatives were involved through a trial steering committee, they gave their thoughts on the study design and development of the topic guide, the manuscript has been amended in the Methods section to include this aspect.
5. Socioeconomic data has been added to table 1 as recommended.
6. We have opted against illustrating recruitment to the qualitative stage of the study with a flow diagram as the same information can be expressed through prose. The Results section has been amended to include number of patients and healthcare professionals approached, we did not record the reason given for declining to participate.
7. The implications for practice has been amended to include implications of AI and implications on training as recommended.

Kind regards,

Dr Ketan Patel
